# Effectiveness and cost-effectiveness of a community intervention in enhancing access to care and improving clinical outcomes for depression: a protocol for a cluster randomised controlled trial in India

**DOI:** 10.1186/s13063-024-08236-0

**Published:** 2024-08-28

**Authors:** Abhijit Nadkarni, Yashi Gandhi, Luanna Fernandes, Kedar Mirchandani, Shreyas Kamat, Helen A. Weiss, Daisy R. Singla, Richard Velleman, Chunling Lu, Urvita Bhatia, Bijayalaxmi Biswal, Miriam Sequeira, Ethel D’souza, Kedar Raikar, Vikram Patel

**Affiliations:** 1 Centre for Global Mental Health, Department of Population Health, London School of Hygiene & Tropical Medicine, London, UK; 2 Addictions and Related Research Group, Sangath, Goa India; 3 Department of Infectious Disease Epidemiology and International Health, London School of Hygiene & Tropical Medicine, London, UK; 4 Department of Psychiatry, University of Toronto, Toronto, Canada; 5 Department of Psychology, University of Bath, Bath, UK; 6 Non-Communicable Disease Cell, Directorate of Health Services, Panaji, Goa India; 7 Department of Global Health and Social Medicine, Harvard Medical School, Boston, USA

**Keywords:** Community intervention, Healthy activity program, Depression, Contact coverage, Effectiveness coverage, Cluster RCT, Implementation effectiveness hybrid trial, India

## Abstract

**Background:**

Although depression is the leading cause of disability worldwide, treatment coverage for the condition is inadequate. Supply-side barriers (e.g. shortage of specialist mental health professionals) and demand-side barriers (e.g. lack of awareness about depression) lead to limited availability of evidence-based interventions, poor demand for care, and low levels of adherence to care. The aim of our study is to examine if the addition of a community intervention delivered by community volunteers enhances the population-level impact of an evidence based psychosocial intervention (Healthy Activity Program [HAP]) in routine primary care by increasing demand for HAP and improving HAP adherence and effectiveness.

**Methods:**

A hybrid type 2 effectiveness implementation cluster randomised controlled trial will be implemented in the state of Goa, India. Twenty-eight clusters of villages and their associated public sector health centres will be randomly allocated through restricted randomisation. Clusters will be randomly allocated to the ‘Community Model’ or ‘Facility Model’ arms. All clusters will offer the HAP and clusters in the ‘Community Model’ arm will additionally receive activities delivered by community volunteers (“*Sangathis”*) to increase awareness about depression and support demand for and adherence to HAP. The primary outcomes are Contact Coverage (Patient Health Questionnaire [PHQ-9] score > 4 as a proportion of those screened) and Effectiveness Coverage (mean PHQ-9 score amongst those who score ≥ 15 at baseline, i.e. those who have moderately severe to severe depression) at 3 months post-recruitment. Additional outcomes at 3 and 6 months will assess sustained effectiveness, remission, response to treatment, depression awareness, social support, treatment completion, and activation levels. Economic and disability outcomes will be assessed to estimate incremental cost-effectiveness ratios. Implementation will be evaluated through process data and qualitative data informed by the RE-AIM framework. A minimum of 79488 primary care attenders will be screened for the Contact Coverage outcome, and 588 individuals with PHQ-9 ≥ 15 will be recruited for the Effectiveness Coverage outcome.

**Discussion:**

If effective, our community intervention will have relevance to India’s Ayushman Bharat universal healthcare programme which is scaling up care for depression in primary care, and also to other low- and middle- income countries.

**Trial registration:**

Registered on ClincalTrials.gov (NCT05890222.) on 12/05/2023.

## Introduction

### Background and rationale {6a}

Depression is the leading cause of disability worldwide and accounts for more than a third of disability-adjusted life years (DALYs) attributed to mental and substance use disorders [1]. The COVID pandemic substantially increased the prevalence of depression with an estimated additional 53·2 million cases of major depressive disorder globally (an increase of 27·6%) after the onset of the pandemic [2]. In 2017, 45.7 million individuals had depressive disorders in India, contributing the most (33·8%) to the DALYs related to mental disorders in the country [3].

Depression treatment coverage across the world is inadequate. It ranges from 33% in high-income countries to 8% in low- and lower middle-income countries (LMICs) [4]. Furthermore, coverage of minimally adequate treatment (i.e. treatment levels deemed minimally sufficient for common mental health problems) is lower still, from 23% in high-income countries to 3% in LMICs [4].

In India, the District Mental Health Program (DMHP) was started in 1996 under the Indian National Mental Health Program (NMHP) to decentralise mental health services by integrating them in general health care delivery and at the community level. However, only a third of Indian states have more than 50% of the population covered by the DMHP, and the treatment gap for depression remains high at 85% [5].

Supply-side and demand-side barriers perpetuate this treatment gap. Supply-side barriers include the lack of skilled human resources. In low-resource settings, task-sharing with non (mental health) specialist workers has become one of the most widely implemented innovations to address this barrier [6]. The evidence on task-sharing of psychosocial treatments for depression is robust and indicates high levels of acceptability and effectiveness of brief psychosocial treatments, delivered over 6-12 weeks in 6-10 sessions by non-specialist workers through routine care and community platforms [7]. Demand-side barriers, notably the lack of recognition of the distress associated with depression as being a health problem, which combined with the stigma attached to mental health problems and help-seeking, lead to low levels of demand for and adherence to depression care [8]. This results in both low *contact coverage* (i.e. the proportion of affected individuals who seek help with a formal service provider) and low *effective coverage* (i.e. the proportion of persons receiving care who attain the desired outcomes) for depression.

### Objectives {7}

Through the IMPRESS (*IMP*lementation of evidence based facility and community interventions to reduce the treatment gap for dep*RESS*ion) programme, we aim to increase both the contact coverage and effective coverage of a scaled-up, evidence-based psychosocial treatment for depression (Healthy Activity Program-HAP) [9] and, in doing so, reduce the treatment gap for depression in a cost-effective manner. We will do this by examining if the addition of a community intervention (Community Model) delivered by community volunteers enhances the population-level impact of scaled-up HAP in routine primary care (Facility Model) by increasing demand for HAP and improving HAP adherence and effectiveness. We will conduct an economic evaluation, to estimate the cost-effectiveness of the Community Model in comparison to the Facility Model, and use the estimates of the cost of the delivery of the interventions to model the financial sustainability of scaling it up across India.

1. Our *primary hypotheses* are that the Community Model will be superior to the Facility Model in reducing the treatment gap for depression through

- (1a) Increasing the demand for depression treatment in primary care (contact coverage outcome); and
- (1b) Reducing depressive symptoms at three months post-recruitment amongst those who screen positive for moderately severe and severe depression (effectiveness outcome)

(2) Our *secondary hypotheses* are that the Community Model will be superior to the Facility Model in

- (2a) Achieving a sustained reduction in symptoms of depression;
- (2b) Increasing short-term and sustained remission from depression;
- (2c) Achieving a greater response to HAP;
- (2d) Increasing perceived social support;
- (2e) Achieving higher treatment completion rates; and
- (2f) Increasing patient-reported activation levels.

In addition, we also hypothesize that the Community Model will increase community awareness about depression.

(3) Finally, we hypothesise that the superior outcomes of the Community Model will be mediated through:

- (3a) Increased community awareness about depression (for contact coverage);
- (3b) Greater perceived social support (for effective coverage);
- (3c) Higher treatment completion rates (for effective coverage); and
- (3d) Increased patient-reported activation levels (for effective coverage).

### Trial design {8}

We will conduct a hybrid type 2 effectiveness implementation cluster randomised controlled trial (CRT) [10], with equal allocation of clusters between the two arms. This design combines clinical effectiveness testing alongside implementation testing, thus aiding in better translation of research into practice, and improved and potentially effective implementation strategies. Our implementation evaluation component will be based on the RE-AIM (*R*each, *E*ffectiveness, *A*doption, *I*mplementation, *M*aintenance) framework [11]. This framework, used extensively in implementation research, enables the measurement of a logical flow of outcomes and better facilitation of translation of research findings and focuses on the external validity of research findings [11].

## Methods: participants, interventions, and outcomes

The proposed trial was preceded by a systematic formative research phase described in a separate paper being written up for peer review. This included a review of the evidence base, interviews with various stakeholders, situation analysis, and consultation workshops. The outcome of this formative research was the community intervention (described below) and finalisation of the trial processes (described below).

### Study setting {9}

IMPRESS will be implemented in the state of Goa, west India. The population of Goa is just over 1.6 million people, with relatively high literacy rates (89% women, 95% men) compared with the national average (65% women, 82% men). We will conduct our trial in 28 clusters of villages associated with health centres in the public sector—20 primary health centres (PHCs), six community health centres (CHCs), and two sub-district hospitals (SDHs). These are frontline healthcare facilities forming the foundation of the public health system in Goa and are collectively referred to as health centres in the rest of this protocol. A typical PHC provides outpatient medical services and some inpatient/observation beds. CHCs perform similar roles to PHCs but cover a larger population and provide some specialist services. SDHs form an important link between PHCs/CHCs on one end and district hospitals on the other end. In addition to primary care, SDHs also provide some specialist services. Specialist mental healthcare (through a psychiatrist, psychologist, psychiatric social worker) is provided in some health centres through fortnightly or monthly clinics by the DMHP [12].

### Eligibility criteria {10}

#### Trial clusters

Our a priori definition of the unit of randomisation was a cluster of villages and the health centre where the residents of those villages seek healthcare. We then used the Geographic Information System (GIS) [13] tool to assess the internal validity of our definition by mapping villages to the health centres in which their residents access healthcare. To reduce the risk of contamination between the two arms, a village is included in a particular cluster if (a) 75% of the healthcare-seeking population from that village accesses services at the health centre associated with that cluster, and (b) it has contiguous borders with other villages in the cluster.

We will measure contact coverage in all the 30 available health centres. However, for the effectiveness coverage we excluded two PHCs as no villages met both the aforementioned criteria with regard to those health centres. This was because the people from the villages surrounding these two PHCs access services from a better resourced SDH which is in close proximity. Thus, for the effectiveness coverage outcome, we randomised 28 clusters.

#### Participants

##### Inclusion

For the Contact Coverage outcome, health centre attenders will be eligible if they are:

- Adults (≥ 18 years);
- Residing in the clusters included in the trial; and
- Speak English or one of the local languages (Konkani, Marathi, Hindi).

In addition to these criteria, health centre attenders will be eligible for the Effectiveness Coverage outcome if they:

- Screen positive for moderately severe or severe depression (total score ≥ 15) on the Patient Health Questionnaire-9 items (PHQ-9) [14]

##### Exclusion

For both outcomes, we will exclude potential participants (and refer them to existing services if needed) who meet one or more of the following criteria:

- Patients with significant speech, hearing, or language impairment that interferes with completion of the screening and/or receipt of psychosocial intervention
- Patients who present to the health centre for emergency medical attention
- Patients with active psychotic symptoms

#### Interventionists and supervisors

The HAP intervention will be delivered by existing health care workers from the participating health centres and primarily include medical officers, AYUSH (traditional medicine) practitioners, staff nurses, Reproductive, Maternal, Neonatal, Child Health and Adolescent (RMNCHA) counsellors, auxiliary nurse midwives (ANMs), and multi-purpose health workers (MPHWs). These individuals (called ‘counsellors’ from here onwards) were nominated for the HAP training by the health officers managing the health centres. The community intervention will be delivered by *Sangathis* (which means ‘a companion’ in the local language) who are community members who are respected in their community and have had previous experience working on socio-developmental, livelihood or governmental community programmes.

HAP clinical facilitators are experienced project-employed staff who have acquired relevant clinical competencies in delivering HAP through training and supervised HAP delivery. They will supervise HAP Counsellors throughout the trial. They will also provide HAP in the health centres as back-up if there are sustained gaps in service in the health centres due to unavailability of the trained counsellors or back-up counsellors. Back-up counsellors are project-employed staff; they will be trained lay counsellors who will be responsible for providing HAP in the health centres in situations where there is a sustained gap in service due to unavailability of the counsellor from the health centre. This will be a backup arrangement for gaps in service occurring due to a variety of reasons such as transfer of the counsellor or when a counsellor goes on leave. *Sangathi* facilitators are project-employed staff who will supervise the *Sangathis*. They are recruited based on their interest to engage in community work and ability to understand and address contextual challenges as well as to network within the community.

### Who will take informed consent? {26a}

All consent procedures with patients will be implemented by trained case managers, adhering to standard ethical guidelines. These project-employed staff will be based in all the health centres. They will be responsible for screening of patients visiting the health centre to identify individuals with depression (the primary implementation outcome). They will also be responsible for scheduling the first HAP session, managing the counsellors’ case load, and referring patients to existing services based on a pre-defined protocol (Table 1).

**Table 1:** Interventionists, supervisors, and support staff

**Staff category**	**Key role(s)**	**Trial arm(s)**
Counsellor	Delivery of HAP	Community Model and Facility Model
Case manager	• Screen patients for depression • Schedule the first HAP session • Manage the counsellors’ case load • Refer relevant patients to existing services such as the DMHP	
HAP clinical facilitators	• Train and supervise counsellors • HAP delivery if there is sustained unavailability of counsellor	
Backup counsellors	HAP delivery if there is sustained unavailability of the primary counsellor	
*Sangathi*	Delivery of community intervention	Community Model
*Sangathi* facilitator	• Support the *Sangathis* in completing the e-course • Address challenges on the field • Collect process data related to community intervention	

The consenting process will be audio-recorded and reviewed by supervisors for quality control purposes. For the contact coverage outcome, we will seek verbal consent from the participants as the outcome will be derived from the screening data and will not be linked to individual identifiers. Individual participant written consent (witnessed thumb impression for illiterate participants) for the effectiveness coverage component of the trial will be obtained from those who are eligible to enrol in this component of the trial.

### Additional consent provisions for collection and use of participant data and biological specimens {26b}

This is not applicable as there are no ancillary studies associated with the trial.

## Interventions

### Explanation for the choice of comparators {6b}

Participants in the control arm (Facility Model) will be offered the HAP, an evidence-based brief psychological treatment for depression. Participants in the intervention arm (Community Model) will be offered an additional community intervention designed to enhance the uptake of and engagement with the HAP.

### Intervention description {11a}

#### Facility model

The HAP, a manualised and evidence-based psychosocial treatment based on behavioural activation [9, 15, 16], will be delivered by the trained counsellors. HAP includes the following strategies: psychoeducation, behavioural assessment, activity monitoring, activity structuring and scheduling, activation of social networks, and problem-solving. Homework is assigned between sessions. HAP will be delivered in an individual format. It entails three phases of treatment, delivered over a maximum of six to eight weekly sessions, each lasting up to 40 min. Sessions will be delivered face-to-face, at the health centre where the counsellors are already employed.

#### Community model

In addition to the Facility Model, village clusters in this arm will receive a community intervention delivered by the *Sangathis* to (i) enhance demand for the HAP treatment and (ii) promote engagement with, and completion of, the HAP treatment. The community intervention is co-produced through a rigorous intervention development process, with local community members, and includes strategies such as activities to increase awareness about depression (e.g. organising community meetings and street plays) and dissemination of psycho-educational materials (e.g. leaflets and posters), identification of people with possible depression in the community, and facilitation of access to HAP (e.g. accompanying them to the health centre for counselling sessions). Additionally, the *Sangathis* will coordinate continuing care of people receiving HAP, through home visits to encourage behavioural activation, homework completion and following up with the patient, and engaging family members to support the patient in achieving treatment goals.

#### Training and supervision

The counsellors received five days of face-to-face or online training on general counselling skills, HAP treatment-specific skills and the supervision process. A 26-item multiple choice questionnaire (MCQ) was administered pre- and post-training to measure knowledge acquisition. Counsellors’ skills to deliver the HAP treatment were assessed using role-plays rated using the EQUIP (Ensuring Quality in Psychological Support) competency assessment tool [17]. After the competency assessment, all the trained counsellors moved on to the ‘internship phase’ where they completed HAP delivery for two people with depression, under the supervision of the HAP clinical facilitator.

*Sangathis* were trained via an e-course covering content related to depression, ways to organise community awareness events, skills for engaging and following-up with community members having depression, and taking care of oneself. The course is divided into five modules, with 4–14 videos in each module. The *Sangathis* were encouraged to complete the course in 4 to 6 weeks. The e-course was supplemented by a handbook that consists of similar content, for quick reference. Pre- and post-training knowledge and attitudes were assessed using MCQs on the content of the course. *Sangathis* were supported to complete the course by a ‘*Sangathi* Facilitator’ who provided coaching over the phone and/or in-person.

Supervision of the counsellors includes (a) peer group supervision [18], virtually once a week, and (b) individual supervision in person once a month. Group supervision is facilitated through a digital app called PEERS (*P*romoting *E*ffective mental healthcare through pe*ER S*upervision). PEERS is a dedicated group supervision mobile app to register patients, collect session-related clinical data, audio record HAP sessions, track the patients’ progress through treatment, and facilitate measurement-based peer supervision by recording the quality rating of the audio-recorded HAP sessions by counsellors.

*Sangathis* are supervised by *Sangathi* facilitators who are responsible for encouraging the *Sangathis* to complete the e-course and supporting them through (a) fortnightly individual meetings to collect process data on the intervention activities conducted; (b) one-to-one support to ensure the quality of the intervention being delivered and resolving challenges they may have faced; and (c) monthly group meetings to share learnings, discuss common challenges, and brainstorm solutions. Table 1 summarises the various individuals involved in delivering, supporting, and supervising the interventions in the trial.

### Criteria for discontinuing or modifying allocated interventions {11b}

We will discontinue treatment in participants who (a) report any serious adverse events (SAE) deemed possibly caused by the trial interventions and (b) refuse treatment after consenting. Such participants will receive a supported referral to the DMHP. Participants who develop serious suicidal ideation or risk of suicidal behaviour will receive supported referral to the DMHP to supplement the HAP. Additionally, participants who do not respond to the treatment at the end of eight sessions of HAP will also receive a referral to the DMHP. All these participants will be contacted for the outcome assessments, unless they withdraw their consent for follow-up.

### Strategies to improve adherence to interventions {11c}

Adherence to the community intervention will be enhanced through the support and supervision provided to the *Sangathis* by the *Sangathi* facilitators as described above.

### Relevant concomitant care permitted or prohibited during the trial {11d}

No concomitant care provided by anyone outside the trial team will be prohibited. Some examples of potential concomitant care are antidepressants prescribed by the medical officers in the participating health centres, the DMHP team or private practitioners, interventions for coexisting physical health problems, and interventions targeting wider social determinants of health (for example, livelihood support). Wherever possible, we will document these other forms of interventions to allow for sensitivity analysis if needed.

### Provisions for post-trial care {30}

There are no provisions for post-trial care as all individuals who receive HAP as a part of the trial would either have completed treatment, dropped out of treatment, or been referred to the DMHP by the end of the trial, and hence continued treatment would not be required.

### Outcomes {12}

Most of the outcomes described here are organised under the *R*each, *E*ffectiveness, *A*doption, *I*mplementation, and *M*aintenance dimensions of the RE-AIM framework. Reach includes the absolute number, proportion, and representativeness of individuals who are willing to participate in a program; Effectiveness covers the impact of an intervention; Adoption includes the absolute number, proportion, and representativeness of settings and people who deliver the program; Implementation refers to the intervention agents’ fidelity to the various elements of an intervention’s protocol, including the cost of the intervention, and the patients’ use of the intervention strategies; and Maintenance includes the extent to which a program becomes institutionalised, and at the individual level, it includes the long-term effects of a program on outcomes [19]. The primary and secondary outcomes represent the Effectiveness dimension, the 6-month outcomes represent the Maintenance dimension, and the costs represent the Implementation dimension of the RE-AIM framework. The process indicators also represent the various dimensions of RE-AIM as indicated in Table 3.

#### Contact coverage (primary outcome)

The contact coverage outcome (PHQ-9 score > 4) will be documented directly through screening in the health centres. After confirming the village of residence of the screened individual, the contact coverage outcome will be allocated to the arm to which their village belongs, regardless of the health centre in which they screen positive.

The contact coverage population level outcome data will be collected through the period of the trial at all thirty health centres, i.e. even at the two health centres which will not be randomised. This is because, while these two health centres do not have discrete village clusters associated with them (as described earlier), individuals with depression from other randomised village clusters could potentially access depression care in these health centres and hence will contribute to the contact coverage outcome.

#### Effectiveness and cost-effectiveness of the integrated intervention

Individual level outcome data will be collected at 3 months and 6 months post-recruitment for those eligible for the effectiveness coverage outcome. The second primary outcome will be mean PHQ-9 score at 3 months post recruitment. The primary endpoint for effectiveness of the integrated intervention is 3 months post recruitment, as we would expect the optimal effect of HAP at that timepoint. The 6-month endpoint is included to evaluate the sustainability of the effect of HAP.

The outcome assessment measures are summarised in Table 2. Depression awareness will be measured using a bespoke tool designed based on the content of the community intervention. The tool will be administered to a consecutive sample of 40 attenders at all the health centres on randomly selected days at baseline and at 6 and 12 months of implementation. Out-of-pocket costs for receiving care, and related non-medical costs, will be collected using a survey tool developed for the purpose which incorporates the Client Service Receipt Inventory (CSRI), a tool that has been used extensively in the study setting [15, 16, 20]. Out-of-pocket costs for receiving HAP intervention (e.g. time loss, travel) will be measured using a survey tool. Standardised disability scores used to estimate quality-adjusted life years (QALYs) will be collected using the WHO Disability Assessment Schedule 2.0 (WHODAS 2.0), which has also been used extensively in this and other settings [16, 21]. The WHODAS assesses behavioural limitations and restrictions related to an individual’s participation, independent from a medical diagnosis [22]. Perceived social support will be measured using a bespoke tool and the Multidimensional Scale of Perceived Social Support (MSPSS) a 12-item instrument designed to measure an individual’s perception of support from family, friends, and a significant other [23]. MSPSS has been widely used and validated in the study setting [24–26]. Additionally, perceived social support related to *Sangathis* will be measured using a bespoke tool. The PREMIUM Abbreviated Activation Scale (PAAS) is a five-item scale, originally developed and used in the trial of the HAP [15, 16] and is based on the Behavioural Activation for Depression Scale (BADS) [27]. It includes five self-reported indicators of behavioural activation such as engagement with a variety of activities and associated pleasure and mastery.

**Table 2:** Outcome evaluation

**Outcome**	**Tool**	**Description**	**Population**	**Timeline**
*Primary*				
Contact coverage	PHQ-9	PHQ-9 score > 4 as a proportion of those screened	Adult health centre attenders	Until we achieve target sample size
Effectiveness coverage	PHQ-9	Mean PHQ-9 score	Individuals who consent to receive HAP	3 months post recruitment
*Secondary*				
Sustained effectiveness	PHQ-9	Mean PHQ-9 score	Individuals who receive HAP	6 months post recruitment
Remission	PHQ-9	PHQ-9 score < 10	Individuals who receive HAP	3 and 6 months post recruitment
Response to treatment	PHQ-9	> 50% reduction in PHQ-9 score	Individuals who receive HAP	3 and 6 months post recruitment
Cost-effectiveness	(1) Inventory form for collecting system-level economic costs of delivering interventions (2a) Client Service Receipt Inventory form for collecting costs to patients (2b) Survey form for collecting costs of receiving HAP intervention to patients (3) WHODAS	(1) System-level costs: economic costs in WHO six building blocks for delivering the interventions (2a) Out-of-pocket costs for receiving general medical care and the related non-medical costs (e.g. time loss, travel) (2b) Out-of-pocket costs for receiving HAP intervention (e.g. time loss, travel) (3) Standardised disability scores used to estimate QALYs	(1) Finance records and individuals involved in delivery of the interventions (2a) Individuals who receive HAP (2b) Individuals who receive HAP (3) Individuals who receive HAP	(1) System-level costs: monthly, during the period of implementation (2a) Patient-level costs: 3 and 6 months post recruitment (2b) Patient level costs: 3 months post recruitment (3) 3 and 6 months post recruitment
Depression Awareness Questionnaire	Bespoke tool	Awareness about depression	Adult health centre attenders	Baseline and on randomly selected days at 6 and 12 months of implementation
Perceived social support	MSPSS	Perception of social support received	Individuals who receive HAP	3 and 6 months post-recruitment
	Bespoke tool			3 and 6 months post-recruitment
Treatment completion	Process data	Met treatment goals or completed the maximum number of sessions or were referred to mental health specialists	Individuals who receive HAP	Across the 12 months of implementation
Behavioural activation	PREMIUM Abbreviated Activation Scale	Patient-report activation levels	Individuals who receive HAP	3 and 6 months post-recruitment

#### Qualitative data

These will include data collected using in-depth interviews (IDIs) and focused group discussions (FGDs) with various stakeholders involved in delivering the interventions, individuals with depression, and decision makers and implementors at various levels in the health system as outlined in Table 3.

**Table 3:** Implementation monitoring and evaluation

**Indicator**	**Source**	**When**	**RE-AIM Dimension**^a^
*Patient level*			
Number of HAP patients who were followed up in the community	Process data from *Sangathi* registers	During implementation	R
% eligible for HAP who receive at least one session of HAP (uptake)	Process data from screening register, PEERS app	During implementation	I
% treatment completers	Process data from PEERS app	During implementation	I
Adherence to homework	Process data from PEERS app	During implementation	I
Acceptability of community intervention	In-depth interviews (IDIs) with individuals receiving the intervention	After completion of 6-month outcome evaluation	R
Number of people with possible depression referred by *Sangathis*	Process data from *Sangathi* registers	During implementation	R
*Counsellor level*			
Characteristics of counsellors who agreed to join the programme	Process data from training register	During training	A
Knowledge	Multiple Choice Questionnaire administered to HAP counsellors	Pre- and post-training	I
Acceptability of training	Focused group discussions (FGD) with counsellors and HAP facilitators	Post-training	A
Fidelity of training	Direct observation of HAP facilitators	During training	I
Skill- based competency	ENACT tool to rate skills of HAP counsellors	Pre- and post-internship	I
Therapy quality	PEERS app	During implementation	I
Acceptability of supervision	IDIs/FGDs with counsellors and HAP facilitators	During implementation	A
Number/duration of supervision sessions attended (feasibility)	PEERS app	During implementation	I
Acceptability of HAP	IDIs/FGDs with counsellors	During implementation	A
Fidelity	Audio recording of HAP sessions	10% of audio-recordings by independent evaluators	I
Perceived benefits and barriers of implementing and sustaining programme	IDIs/FGDs with counsellors	During implementation	A
*Sangathi level*			
Characteristics of *Sangathis* who agreed to join the programme	Process data from recruitment register	During training	A
Acceptability of training	IDIs/FGDs with *Sangathis* and *Sangathi* facilitators	Post-training	A
Number of attempts to complete module knowledge tests	Process data from e-course	During training	I
Acceptability of supervision	IDIs/FGDs with *Sangathis* and *Sangathi* facilitators	During implementation	A
Number/duration of supervision sessions attended (feasibility)	Process data from facilitator log	During implementation	A
Acceptability of community intervention	IDI/FGDs with *Sangathis*	During implementation	A
Retention	Process data	During implementation	A
Type and number of community activities conducted	Process data from facilitator log	During implementation	I
Number of people attending community activities	Process data from facilitator log	During implementation	R
Number of *Sangathi* contacts with participants in the effectiveness coverage sample	Process data from facilitator log	During implementation	I
Perceived benefits and barriers of implementing and sustaining programme	IDI/FGDs with *Sangathis*	During implementation	A
Costs borne towards the delivery of the community intervention	Form for collecting costs borne by *Sangathis* and the community	During implementation	E
*Health systems level*			
Perceived benefits and barriers of implementing and sustaining programme	IDI/FGD with health department officials	At the end of implementation	A
Fiscal capacity to cover the costs of scale-up by the government	Macro-level data collection tool	During implementation	I
Readiness to change	ORIC administered to health department officials and other staff from the health centres, e.g. counsellors, and health officers	Baseline and 12 months	A

^a^*R* Reach, *E* Effectiveness, *A* Adoption, *I* Implementation, *M* Maintenance

#### Process indicators, implementation monitoring, and evaluation (Table 3)

These data, informed by the RE-AIM framework, will be collected at the level of the patient, counsellor, *Sangathi*, and health-system. These include data collected routinely as a part of the implementation (e.g. treatment completion rates), qualitative data, and administration of standardised tools. At the level of the patients who receive HAP, we will collect data about treatment initiation (uptake), follow-up rate in the community, homework adherence rate, treatment completion rate, referral from the community, and overall acceptability of the community intervention. At the level of the counsellors, we will collect data related to the characteristics of counsellors, knowledge acquisition through the training, acceptability of training, fidelity of training delivered, acquisition of skill-based competency, quality of therapy delivered, feasibility and acceptability of supervision, acceptability of HAP, fidelity of the HAP delivered, and perceived benefits and barriers of implementing and sustaining the program. At the level of the *Sangathi,* we will collect data related to characteristics of *Sangathis*, acceptability of training, number of attempts to complete module-based questions, feasibility and acceptability of supervision, acceptability of community intervention, retention in the program, type and number of community activities conducted, number of people attending community activities, number of *Sangathi* contacts with patients, and perceived benefits and barriers of implementing and sustaining the program. Finally, at the level of the health system, we will collect data from various stakeholders (e.g. health department officials) about perceived benefits and barriers of implementing and sustaining the program, the fiscal capacity to cover the costs of scale-up by the government, and readiness to change using the Organizational Readiness for Implementing Change (ORIC) tool. The ORIC has nine items that measure organisational readiness to change in two domains, change commitment and change efficacy [28].

#### Contamination

Residents in the Facility Model clusters could be exposed to the community intervention (e.g. flyers, community events). Such contamination could potentially bias the trial results towards the null, reducing the estimated effect of the community intervention. We will mitigate the effect of potential contamination through our design (limiting the geographical contiguity of clusters), by estimating potential contamination, and through statistical analysis. We will estimate contamination at baseline by asking those who are screened whether they have been exposed to any of the components of the community intervention (e.g. attended awareness events, saw flyers). These data will be used to conduct sensitivity analysis estimating the contamination range and the actual treatment effect.

### Participant timeline {13} (Table 4)

**Table 4:**
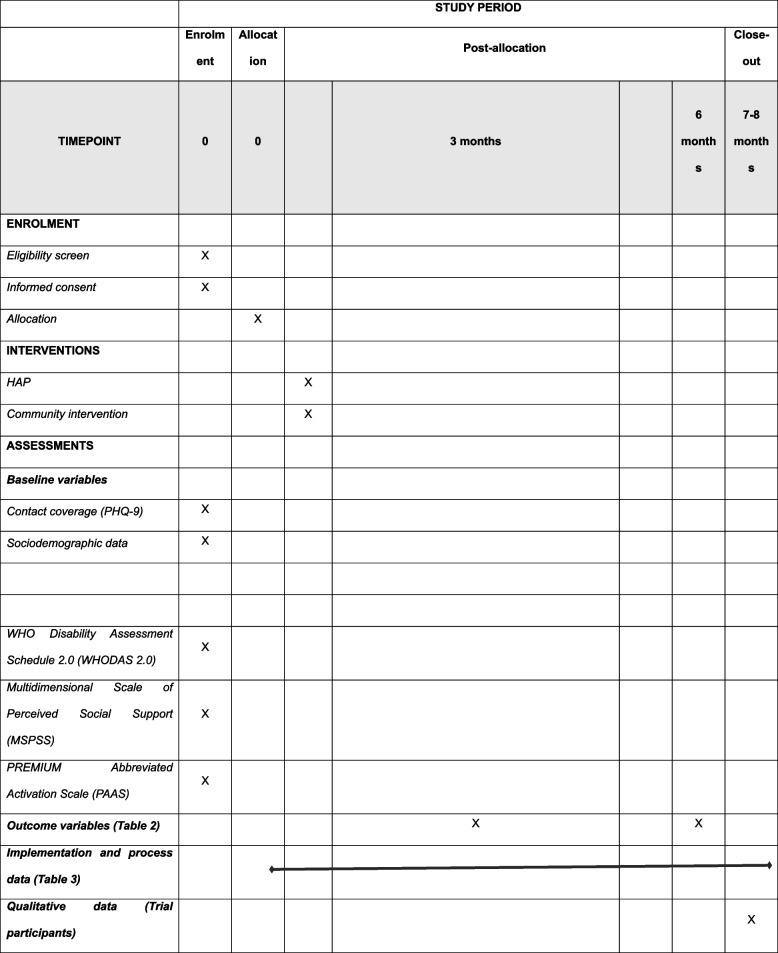
Schedule of enrolment, interventions, and assessments

Participants will be identified and recruited by the trained and supervised case managers at the health centres. Health centre attenders meeting the eligibility criteria will be screened using the PHQ-9 questionnaire [29], a nine-item questionnaire that has been validated and extensively used in the study setting [15, 30]. Participants who score > 4 (mild depression and above) will contribute to the contact coverage outcome.

Due to the limited caseload capacity of the counsellors, we will prioritise HAP for the most severely unwell, who have also been shown to derive the greatest benefit from such interventions [31]. This means that only those who score ≥ 15 (moderately severe to severe depression) will be eligible to receive the HAP intervention and will contribute to the effectiveness coverage outcome. After consent has been obtained from those eligible for the effectiveness coverage component, the case manager will collect the data on the following potential effect-moderators: age, gender, marital status, education and employment status; and other outcomes: WHODAS 2.0, MSPSS, and PAAS. All assessments will be audio-taped (with consent), and the tapes will be randomly selected for review by the supervisor for quality assurance. The outcomes will be measured at 3 and 6 months post recruitment. The qualitative data with trial participants will be collected after the 6-months outcome evaluation. The process data will be collected at various timepoints during the implementation as outlined in Table 3.

### Sample size {14}

#### Contact coverage outcome

We will screen for 5 days a week for 12 months (52 weeks) in all 30 health centres. Based on screening numbers over the first two months of the trial (average of 6624 per month), we will be able to screen 79,488 over 12 months. Based on the screening data from the first 2 months of the IMPRESS trial, the prevalence of depression (PHQ-9 score > 4) amongst those screened at the health centres is 14%. Assuming this prevalence in health centres in the Community Model, this sample size provides 86% power to detect a difference with a prevalence of 11% in those screened at the health centres in the Facility Model at the 5% significance level, assuming a coefficient of variation of 0.2 as below.

#### Effectiveness coverage outcome

Twenty-one participants with a PHQ-9 score ≥ 15 will be recruited from each of the 28 clusters (*N* = 588). Allowing for 20% of participants to be either not traceable by the outcome assessors or to not consent to participate at follow-up, we expect 17 participants from each cluster to complete the outcome evaluation at the three-month endpoint. This gives a sample size of 476 in which to assess our effectiveness coverage outcome at three months. This sample size provides 80% power to detect an effect size (standardised mean difference (SMD)) of 0.4 at the 5% significance level, assuming a coefficient of variation between clusters of 0.2. The assumptions underlying this calculation are based on the HAP trial for the effect size (i.e. a difference between the mean PHQ-9 scores of 11.9 [SD = 7.0] in the control arm and 8.9 [SD = 7.2] in the intervention arm) and the MANAS trial for the coefficient of variation (this trial tested a psychosocial intervention for common mental disorders in the same setting [32, 33]).

### Recruitment {15}

We will continue screening in the 30 health centres in Goa, India, until we achieve the sample size mentioned above. Based on our experience of implementing trials in this setting, we expect to achieve the planned sample size over 12 months of screening and recruitment.

## Assignment of interventions: allocation

### Sequence generation {16a}

The allocation sequence was generated through restricted randomisation using the following parameters: (a) type of health centre (PHC, CHC, SDH) associated with the cluster; (b) availability of DMHP services in the health centre; and (c) population size of the cluster. A randomly selected seed number was used to enable the randomisation procedure to be reproduced. The randomisation was done before the start of the trial as both the embedding of the trial and other implementation processes (e.g. training of community intervention delivery agents) had to be initiated in advance of recruitment of the first participant.

### Concealment mechanism {16b}

An independent statistician conducted the randomisation procedure and communicated the allocation of clusters only to the data manager and community intervention coordinator to initiate relevant procedures in the Community Model clusters (e.g. identification of *Sangathis*). The coordinator will share the information only with those members of the team who would naturally remain unblinded because of the nature of their role (e.g. *Sangathi* facilitators).

### Implementation {16c}

The enrolment of participants will be done by case managers. All consenting participants in the Facility Model will receive HAP, and those in the Community Model will receive support from the *Sangathi* in addition to the HAP.

## Assignment of interventions: blinding

### Who will be blinded {17a}

Throughout the trial, the lead principal investigators and co-investigators, programme director, research coordinator, outcome team coordinator, outcome assessors, and trial statistician will be blinded to arm allocation. Given the nature of the interventions and the roles, the following will be unblinded: participants, counsellors, *Sangathis*, case managers, HAP facilitators, *Sangathi* facilitators, data operators and data manager, and clinical coordinators.

Blinding will be maximised by:

- Allocating unique participant IDs which have no association with the cluster and arm identity
- Allocating a random alphabet as an ID to clusters
- Ensuring that the outcome assessors’ team is separate from the rest of the team, comes to office only on weekends and is not privy to the randomisation allocation
- Emphasising to assessors that all patients are receiving an intervention (not specifying whether this is only HAP or HAP plus the community intervention) and that there is genuine equipoise about which is better
- Administering the PHQ-9 (the primary outcome) prior to all other outcome and qualitative assessments
- Ensuring that process and qualitative research evaluations are carried out independent of the quantitative evaluations; the qualitative research will be carried out by a separate team only after the quantitative evaluation is complete

### Procedure for unblinding if needed {17b}

No procedure for unblinding is planned for this trial.

## Data collection and management

### Plans for assessment and collection of outcomes {18a}

The individual level effectiveness outcomes will be assessed at the participants’ homes, any other convenient location, or over the phone by a team of researchers independent of the intervention team and blind to the allocation status of the participant. The primary endpoint assessment will occur within 4 weeks of the 3-month endline date and similarly for the 6-month endline. The qualitative data will be collected by a team of researchers which is independent from the outcome evaluation team and treatment providers.

### Plans to promote participant retention and complete follow-up {18b}

All participants will be informed about the importance of timely follow-up and of their contribution during the consenting process. For each of the two follow-ups, there will be a 4-week window to maximise the chances of completing the outcome assessments. As there is a possibility that some participants are unable to complete the assessments within this window, we will not reject any outcome assessments done outside this window, and we will conduct a sensitivity analysis after excluding any such assessments. Whenever we have issues with contacting the participant, we will make three attempts using different modalities (e.g. phone call, home visit) at different times of the day on different days of the week to maximise the chances of completing the assessment.

### Data management {19}

All screening, baseline, and outcome data will be collected and managed using REDCap, a secure, web-based software platform for data collection in research studies. All members of the team involved in data collection are trained about the study procedures to ensure consistent and reliable data collection. All the digital data (including audio-recordings) will be saved on a secure institutional server and backed up to a secure cloud server on a weekly basis. Access to pre-locked data will be password-protected at multiple levels, and no member of the trial team apart from the data team and statistician will have access to these passwords. The PIs will have access to all the anonymised trial data and will decide about the access privileges to the database. The data manager and the data team working under her supervision will be responsible for the following activities: (1) development of the data capture tools in REDCap, (2) data monitoring and quality control, and (3) data processing. The data team will prepare regular and blinded reports for monitoring of progress toward trial milestones. While the REDCap data entry system reduces data errors (for example, by not accepting out-of-range values), the data team will also conduct manual checks for the accuracy of the data. This will include checking for duplicate records, illogical entries, outlier values, and out of range values. As people are now recognisable by their voices, all audio recordings of interviews and counselling sessions will be deleted after transcription and analysis of the data. All paper data, including the signed consent forms, will be converted into digital files, and saved on the secure institutional server.

### Confidentiality {27}

All research staff will be trained in Good Clinical Practice guidance. All participant-identifying information on paper (e.g. signed consent forms) will be kept in locked cabinets accessible only to the data team. All electronic data will be password protected. The data obtained during the trial will be used only for the purpose for which the participant has consented. Each study participant will receive a unique identification number to which all their trial data will be linked. Audio files of recruitment, follow-up, and qualitative interviews will also be renamed using the corresponding participant identification number. The file that links participant names with the participant identification number will be kept in a single locked cabinet, accessible only to the data manager. Finally, all data will be reported only in an aggregate form.

### Plans for collection, laboratory evaluation, and storage of biological specimens for genetic or molecular analysis in this trial/future use {33}

This is not applicable as this trial does not involve collection of any biological specimens for genetic or molecular analysis.

## Statistical methods

### Statistical methods for primary and secondary outcomes {20a}

A detailed analysis plan will be agreed with the data safety and monitoring board towards the end of the trial and before any analysis is undertaken. Findings will be reported as per the CONSORT guidelines for CRTs. Baseline comparability will be assessed for participants who could and could not complete outcome assessments. Comparability of the two intervention arms will be assessed for potential confounding factors such as age, gender, education, and severity of depression at baseline.

#### Contact coverage

The primary analyses will compare the proportion of individuals identified with depression through screening in the health centres. Logistic regression will be used to compare the number of individuals between arms, allowing for within-facility clustering. We will also do a sensitivity analysis to examine the contact coverage for moderate depression and above (i.e. PHQ9 ≥ 9).

#### Effectiveness coverage

The primary analyses will be intention-to-treat. Linear regression GEE will be used to compare mean PHQ-9 scores by arms, adjusting for within-facility clustering, baseline severity of depression and any confounding factors for which there is substantial imbalance between the arms at baseline. We will present an estimate of the effect size as an adjusted SMD and 95% confidence intervals and the intra-cluster correlation. For binary outcomes, the effect will be measured by the risk ratio (RR) and 95% confidence intervals using logistic regression models adjusted for within-facility clustering. Predefined effect-modification analyses will include adjustment for age, gender, marital status, education, employment status, and baseline PHQ-9 score. Finally, we will consider a sensitivity analysis to investigate the effect of missing data by using multiple imputation. We will not conduct an interim analysis as we do not anticipate any safety concerns related to the interventions.

#### Economic evaluation

This will be based on the estimation of the actual costs of delivery of the Facility and Community Models and costs incurred by individual participants, as described in Table 2. To generate incremental costs of the two models, we will use multi-level generalised linear models (and controlling for clustering effects for skewed cost data) at the individual patient-level analysis. Costs per patient will be the outcome variable and one dummy variable indicating the two models (with the Facility Model as the reference) will be the exposure variable. Other covariates include time and interaction terms between intervention dummies and time. To provide mean and 95% confidence intervals (CIs) for the estimated incremental costs, we will use bootstrapping. The same strategy can be applied to incremental effectiveness and incremental cost-effectiveness ratios (ICERs) for generating their means and 95% CIs. We will generate cost-effectiveness acceptability curves with different levels of thresholds on willingness to pay, from either health system perspective or societal perspective, to show the probability that the intervention remains cost-effective as thresholds change. We will also specifically examine the extent to which the interventions impact on out-of-pocket expenditure for health care, one of the leading causes of health-related impoverishment for people with depression. We will assess the long-term financial sustainability of the scaled-up Community Model by estimating the costs of implementation of the Community Model (which includes the HAP) at the national level. We will estimate the fiscal capacity for scaling up the model at a regional and national level.

### Interim analyses {21b}

There are no plans for any interim analyses because the safety and effectiveness of the HAP intervention has been demonstrated in a previous trial.

### Methods for additional analyses (e.g. subgroup analyses) {20b}

#### Mediation

We will conduct mediation analysis using the Monte Carlo Method for Assessing Mediation (MCMAM) to assess the effects of quantitatively measured a priori mediators on primary outcomes [34]. MCMAM performs better than the Sobel test and comparably with bootstrapping approaches [35]. After examining individual mediation pathways using MCMAM, we will conduct a multiple mediation model examining all significant mediation pathways using structural equation modelling (SEM). All analyses will adjust for relevant baseline variables as well as potential covariates, including patient-, counsellor-, and treatment-level characteristics [36].

#### Qualitative

A thematic analysis approach will be used to analyse qualitative data [37]. The themes used for the interviews and group discussions will provide the a priori framework for mixed inductive-deductive coding of the data. All FGDs and IDIs will be audio-taped; the recorded interviews will be transcribed verbatim and local language interviews will be translated into English. Subsequently, the memos (reflective notes about the interviews) and field notes written by the interviewers will be attached to the main text of the interviews. Data collection and analysis will progress iteratively, identifying and interpreting themes, leading to modifications to the interview and group discussion guides. Coding will involve grouping participants’ responses into categories that bring together similar ideas, concepts, or themes. Patterns will be derived by comparing responses to themes and examining how themes are interacting with each other.

### Methods in analysis to handle protocol non-adherence and any statistical methods to handle missing data {20c}

Outcome and covariate data will be investigated for missingness, and multiple imputation methods will be considered.

### Plans to give access to the full protocol, participant-level data, and statistical code {31c}

The full protocol is available on ClinicalTrials.gov (ID NCT05890222). One year after unmasking and completion of analyses of both 3- and 6-month outcome data (whichever is earlier), the trial dataset will be stored in a publicly available repository. The stored dataset will only have anonymised participant data.

## Oversight and monitoring

### Composition of the coordinating centre and trial steering committee {5d}

Two committees will monitor the progress of the trial (Table 5). Summary statistics and graphs showing trends over time will be compiled for the process indicators and reported on a three-monthly basis to the trial steering committee.

**Table 5:** Trial management committees

**Committee**	**Role**	**Members**	**Frequency of meetings**
Trial management committee (TMC)	• Monitor all aspects of the conduct and progress of the trial • Ensure that the protocol is adhered to • Take appropriate action to safeguard participants and the quality of the trial	• Lead principal investigator • Programme director • Intervention team leaders • Project coordinators • Data manager	Fortnightly
Trial steering committee (TSC)	• Overall supervision of the trial • Approval of trial protocol and any protocol amendments • Ensure compliance with protocol • Final decisions about continuation or termination of the trial or substantial amendments to the protocol	• Principal investigators (chairperson) • Co-investigators • Programme director • Intervention team leaders • Project coordinators	Three-monthly

### Composition of the data monitoring committee, its role and reporting structure {21a}

The data safety monitoring board (DSMB) will meet before the start of the trial and at 6 monthly intervals. It will be independent of the sponsor and competing interests. It will review the accruing trial SAE reports, determine whether there are any safety issues that should be brought to participants’ attention, determine if there are any reasons for the trial not to continue or pause, and make recommendations to unblind data and make further recommendations to the TSC. It comprises of a psychiatrist with expertise in community trials (Professor Pallab Maulik), a public health expert with expertise in community interventions (Dr Tanya Sheshadri), a bioethicist (Dr Neha Chawla), and a biostatistician (Dr Nikhil Gupte).

### Adverse event reporting and harms {22}

We expect serious adverse events (SAEs) to be reported by the participant, observed by the counsellors and/or *Sangathi* during intervention delivery and by outcome assessors during the 3- and 6-month outcome evaluation. The following serious adverse events (SAEs) will be reported to the DSMB and IRBs as per their guidelines: unplanned hospitalisation, attempted suicide, victimisation (violence against the trial participant), and death by any cause, including suicide. The report will also be shared with a clinician independent of the trial. The independent clinician will contact the participant to complete a detailed interview to determine any association with trial interventions and recommend any necessary intervention. No stopping rules are proposed because SAEs are not expected to result in the trial as the HAP intervention is not experimental or associated with SAEs.

### Frequency and plans for auditing trial conduct {23}

The study sponsor is responsible for organising monitors to review the trial documents as needed, to determine whether the data reported are complete and accurate. The sponsor selects projects to audit using a risk-based approach, overseen by the research governance committee. During the sponsor review process, the research facilitator will conduct an organisational risk assessment to determine a risk ranking for the project. If selected, the auditor will review the overall quality and completeness of the data, examine source documents, interview investigators and coordinators, and confirm that the trial has complied with the protocol. The auditor will verify that all adverse events were documented in the correct format and are consistent with the protocol.

### Plans for communicating important protocol amendments to relevant parties (e.g. trial participants, ethical committees) {25}

Protocol amendments will be discussed and agreed upon by the investigators and subsequently be implemented only following approval by the relevant ethics committees. Protocol amendments will also be reported to IRBs and DSMB and updated in the clinical trial registry.

### Dissemination plans {31a}

The trial protocol has been registered on ClinicalTrials.gov. The findings from the trial will be submitted for publication in international, peer-reviewed journals. Findings will be shared with key stakeholders (e.g. Ministry of Health and Family Welfare, Directorate of Health Services, trial clusters). Other outputs will include presentation of findings at relevant national, regional, and international scientific conferences.

## Discussion

While there is established evidence on the effectiveness of task sharing and psychosocial interventions for depression in developing countries, questions persist about how these can be scaled up effectively in routine primary care while enhancing uptake and impact. The IMPRESS trial will extend the evidence on the HAP intervention and how a potentially low-cost community intervention can enhance the access to and effectiveness of an evidence-based low-intensity psychosocial intervention for depression. While the trial addresses the leading cause of mental health-related global burden of disease, it does so by leveraging existing and affordable resources in primary care and communities.

As these factors are critical for scalability of interventions, especially in low resource settings, we expect our findings to be of particular interest to policymakers. The findings from our trial are expected to inform policy makers in developing countries on the practical implementation of an evidence-based psychosocial intervention for depression (and other similar interventions) in low-resource settings using existing human resources in primary care and augmented by community volunteers. We also expect our findings to be generalisable to other developing countries because our implementation intervention strategies emphasise acceptability, affordability, utilisation of locally available resources, and deployment of methods to address barriers to scaling up psychosocial interventions in low resource settings.

One component of our implementation strategy might appear to be non-generalisable—screening, that too using a dedicated ‘case manager’. Ideally, one would expect that individuals with depression would be identified and referred for psychosocial intervention by the primary care physician. However, there is substantial evidence that demonstrates that the recognition rates for depression are extremely low in routine primary care practice in LMICs [38] and training of existing primary healthcare workers does not lead to a sustainable increase in identification of depression in primary care [39]. Hence, for mental health programmes based in primary care settings, policymakers will need to consider supporting additional resources necessary for effective scale-up, and our trial will provide the first systematic evidence of this kind from a LMIC.

Ultimately, we anticipate that the evidence generated by IMPRESS will contribute to reducing the treatment gap for depression in low-resourced settings through delivering an evidence based psychosocial intervention in routine care and a range of practical implementation tools (such as the community intervention) to enhance the impact of the intervention. Considering the limited resources allocated to mental healthcare even in high-income countries and the high costs of specialist human resource in such countries, one would expect such interventions to potentially have applicability even in these countries.

In summary, IMPRESS is, to the best of our knowledge, the first systematic attempt in any LMIC to scale up an empirically supported psychosocial treatment in primary care and integrating this implementation with a community intervention to enhance its contact and effective coverage. In doing so, IMPRESS will address one of the major unanswered challenges in global mental health, i.e. the methods, acceptability, effectiveness, and cost-effectiveness of integrated community and primary care-based approaches to reduce the treatment gap for depression. In India, IMPRESS is timely as it aims to evaluate a model of a scaled-up integrated mental health care which is aligned with the Government of India’s ambitious Ayushman Bharat programme [40] which aims to make affordable and quality health services, including mental healthcare, accessible geographically by integrating them into primary health services and the community.

## Trial status

The start date of the trial was 1 November 2023, and the anticipated end date for recruitment is the 31 October 2024. Protocol version 8 at 20.05.2024.

## Data Availability

The joint lead investigators (AN, VP) will have access to the final trial dataset. After publication of the primary and secondary trial findings, the trial datasets will be made available through a data repository.

## Abbreviations

ANM: Auxiliary nurse midwives
BADS: Behavioural Activation for Depression Scale
CHC: Community health centres
CI: Confidence interval
CRT: Cluster randomised controlled trial
CSRI: Client Service Receipt Inventory
DALYs: Disability-adjusted life years
DHS: Directorate of Health Service
DMHP: District Mental Health Program
DSMB: Data safety and monitoring board
FGD: Focussed group discussion
GEE: Generalized estimating equations
GIS: Geographic Information System
HAP: Healthy Activity Program
ICER: Incremental cost-effectiveness ratios
IMPRESS: IMPlementation of evidence-based facility and community interventions to reduce the treatment gap for depression
IDI: In-depth interview
LMICs: Low- and middle-income countries
MCMAM: Monte Carlo Method for Assessing Mediation
MPHW: Multi-purpose health workers
MSPSS: Multidimensional Scale of Perceived Social Support
NMHP: National Mental Health Program
ORIC: Organizational Readiness for Implementing Change
PAAS: PREMIUM Abbreviated Activation Scale
PEERS: Promoting Effective mental healthcare through peER Supervision
PHC: Primary health centres
PHQ-9: Patient Health Questionnaire-9 items
QALY: Quality-adjusted life year
RE-AIM: Reach, Effectiveness, Adoption, Implementation, Maintenance
RMNCHA: Reproductive, Maternal, Neonatal, Child Health and Adolescent
RR: Risk ratio
SAE: Serious adverse event
SDH: Sub-district hospital
SEM: Structural equation modelling
SMD: Standardised mean difference
TMC: Trial management committee
TSC: Trial steering committee
WHODAS: WHO Disability Assessment Schedule
